# A study protocol for a randomized controlled trial of a cross-systems service delivery model to improve identification and care for HIV, STIs and substance use among justice-involved young adults

**DOI:** 10.1186/s40352-020-00121-7

**Published:** 2020-08-14

**Authors:** Katherine S. Elkington, Megan A. O’Grady, Susan Tross, Patrick Wilson, Jillian Watkins, Lenore Lebron, Renee Cohall, Alwyn Cohall

**Affiliations:** 1 Department of Psychiatry, Columbia University and the New York State Psychiatric Institute, New York, NY USA; 2 Clinical Medical Psychology, HIV Center of Clinical and Behavioral Studies, 1051 Riverside Drive, #15, New York, NY 10032 USA; 3 University of Connecticut, School of Medicine Department of Public Health Sciences, 263 Farmington Avenue, Farmington, CT 06030-6325 USA; 4 Mailman School of Public Health, Columbia University, New York, NY USA; 5 Center for Court Innovation, New York, NY USA; 6 New York Presbyterian Hospital, New York, NY USA

**Keywords:** HIV/STI testing, SU screening, Justice-involved, Young adults, Service linkage

## Abstract

**Background:**

Justice-involved young adults (JIYA) aged 18–24 are at significant risk for HIV and problematic substance use (SU) but are unlikely to know their HIV status or be linked to HIV or SU treatment and care. Intensive efforts to increase screening and improve linkage to HIV and SU services for JIYA are needed that address youth as well as justice and health/behavioral health system-level barriers.

**Methods:**

MoveUp is a four-session intervention that integrates evidence-based protocols to promote HIV and STI testing, HIV and SU behavioral risk reduction and engagement in treatment for JIYA. MoveUp is delivered onsite at an alternative sentencing program (ASP) by HIV testing outreach workers from a youth-focused medical and HIV treatment program. *N* = 450 youth are randomized following baseline assessment into two groups: MoveUp or standard of care. Youth are followed for 12 months following the intervention; unprotected sexual behavior, substance use, HIV and STI testing as well as treatment linkage will be assessed at 3, 6, 9 and 12-months.

**Discussion:**

This study is one of the first to systematically test an integrated screen/testing, prevention intervention and linkage-to-care services program (MoveUp), using evidence-based approaches to address the overlapping HIV/STI and substance use epidemics in JIYA by providing on-site services to identify HIV/STI and SU risk and treatment need within justice-settings as well as linkage to services in the community. This approach, capitalizing on health and justice partnerships, represents an innovation that can capitalize on missed opportunities for engaging JIYA in health care.

## Background

HIV prevalence in US justice populations is 2–5 (CDC, 2012b; Martin, O'Connell, Inciardi, Surratt, & Beard, 2003; Maruschak, 2011; New York State Department of Health, 2010; Spaulding et al., 2009) times higher than in the general population, and justice-involved young adults (JIYA) aged 18–24 are at particular risk (Dembo, Belenko, Childs, Greenbaum, & Wareham, 2010; Elkington et al., 2008; Frye, Wallace, Chavez, & Luce, 2008; Joesoef, Kahn, & Weinstock, 2006; Kahn et al., 2005; Morris et al., 1995; Pack, DiClemente, Hook III, & Oh, 2000; Romero et al., 2007). Many JIYA reside in neighborhoods with high community HIV viral loads, exposing them to high risk sexual networks. Recent Adolescent Trial Network (ATN) data found that one in every three HIV+ youth had prior justice involvement (Gamarel et al., 2016). Furthermore, JIYA have higher rates of HIV risk behaviors than general population youth (Elkington et al., 2008; Morris et al., 1995; Pack et al., 2000; Romero et al., 2007), and much higher rates of STIs (4%–48%) (Dembo et al., 2010; Frye et al., 2008; Joesoef et al., 2006; Kahn et al., 2005). With high HIV risk behaviors, and residence in poor, high-prevalence communities, JIYA may be more likely to be HIV+ than general population youth, but are just as likely to be unaware of their status (Van Handel, Kann, Olsen, & Dietz, 2016; Zanoni & Mayer, 2014). Unidentified and untreated – JIYA are at particular risk for acquiring HIV and further contributing to transmission in their communities.

HIV/STI risk in JIYA is further exacerbated by substance use and disorder (SU/D) among this population. A substantial body of literature documents the positive association between substance use (SU) and sexual risk behavior among adolescents and young adults (Bryan, Schmiege, & Magnan, 2012; Shorey et al., 2019; Tucker, Shih, Pedersen, Seelam, & D’Amico, 2019), including reduced engagement in, and adherence to, HIV care (Lucas, 2011). For example, among youth and adults who are HIV+, early onset and problem SU are associated with poor adherence to HIV medications, suboptimal viral load suppression (Arnsten et al., 2002; Comulada, Swendeman, Rotheram-Borus, Mattes, & Weiss, 2003; Cook et al., 2001; Gordillo, del Amo, Soriano, & González-Lahoz, 1999; Hosek, Harper, & Domanico, 2005) and difficulty accessing and remaining in HIV care (Bartlett, 2002; Freudenberg, 2006; Rich et al., 2001; Zaller et al., 2008). JIYA have much higher rates of SUD (25–50%) (Baillargeon et al., 2009; Center for Behavioral Health Statistics and Quality, 2017; Ferguson, Bender, Thompson, Xie, & Pollio, 2012; Smith & Trimboli, 2010; Wasserman, McReynolds, Lucas, Fisher, & Santos, 2002) than non-justice involved young adults (15%–22%) (Center for Behavioral Health Statistics and Quality, 2017; Kessler, Chiu, Demler, Merikangas, & Walters, 2007); studies report that about one-third of arrested JIYA were using alcohol and/or drugs at the time of arrest (Karberg & James, 2005). Taken together, these findings suggest that failure to address SU in JIYA may greatly increase the likelihood that they will acquire or transmit HIV and not engage and remain in HIV care.

Unfortunately, data indicate that JIYA are neither accessing HIV testing (Tolou-Shams et al., 2017; Tolou-Shams, Conrad, Louis, Shuford, & Brown, 2015) the first step to engaging in care, nor SU treatment in the community (Davis, Dumas, Wagner, & Merrin, 2016). In the context of considerable risk factors – chaotic and disconnected families, school dropout, unemployment and high risk neighborhoods – and the lessening of social supports associated with young adulthood (Arnett & Tanner, 2006) these young adults are often disconnected from health and behavioral health services in the community. Thus, the justice system becomes their de facto health service. Yet, despite well-documented HIV, STI and SU needs, and Centers for Disease Control and Prevention (CDC) recommendations for regular testing of high risk populations, such as those who are justice-involved (CDC, 2012a), JIYA do not receive HIV testing (or SU screening) routinely in justice settings (Belenko & Dembo, 2003; Elkington et al., 2016; Taxman, Henderson, & Belenko, 2009; Taxman, Perdoni, & Harrison, 2007; Teplin, Abram, McClelland, Washburn, & Pikus, 2005). National surveys of locked and community correctional facilities found between 18%–31% offered HIV and/or STI testing (Elkington et al., 2020; Hammett, Kennedy, & Kuck, 2007) and about one-third do not screen for SU problems (Taxman, Cropsey, Young, & Wexler, 2007). Without screening and identification, effective intervention and service uptake is significantly limited (Wasserman et al., 2009).

Alternative sentencing programs (ASPs), those that divert court-involved individuals to programming within the community, may be potentially ideal settings in which to identify young adults at high risk for HIV and STIs, and implement programs that link JIYA to HIV, STI, and SU services in the community. These programs have proven successful in allowing young adults to maintain daily goals, activities and supports, while meeting their criminal justice responsibilities. Community supervision represents the largest branch of the justice system, as 1 in 55 people were under community supervision in the US at the end of 2016 (Kaeble, 2018). As such, working in these programs will result in being able to intervene with a large number of hard-to-reach JIYA. However, because such programs may be more focused on community safety and fulfilling court mandates than health, additional resources, staffing, and training would be required to implement HIV/STI screening and prevention.

The complex challenges of improving delivery of health-related services within settings serving JIYA demands innovative strategies to address this service gap. Whereas such settings may inadequately address HIV risk reduction and testing needs, community health agencies are uniquely positioned to provide HIV/STI testing, treatment and prevention programming for JIYA while they are in ASPs. Community health agencies designed for young adults with HIV and SUD are rarely linked to community service programs for JIYA, thus there are missed opportunities for cross-linkages and care coordination. Prior work has shown the utility of collaborations between different service systems to address unmet HIV and related service needs of incarcerated populations (Belenko, Hiller, et al., 2013; Belenko, Visher, et al., 2013; Pearson et al., 2014; Visher et al., 2014). For JIYA, collaboration between the justice and public health systems are essential to increase the likelihood that youth are routinely tested for HIV and other STIs, screened for SU, and linked to necessary treatment and counseling services. Further, studies focused on HIV among JIYA often conclude with the same recommendations: to increase access to HIV/sexual health services and integrate sexual health and SU interventions as part of sentencing and/or community reintegration programs (Elkington et al., 2008; Teplin et al., 2005; Tolou-Shams, Brown, Gordon, Fernandez, & Group, 2007). Providing such integrated services within ASPs may be a highly effective approach to reducing highly interrelated problems, HIV and STI transmission and SU, via identification of risk/treatment need and linkage to services in a single service model.

In addition to addressing system level linkages and service integration within ASPs, better interventions are needed to address HIV and SU among JIYA. Existing HIV prevention interventions for justice-involved youth show variable efficacy (Tolou-Shams, Harrison, Hirschtritt, Dauria, & Barr-Walker, 2019) and are largely developed for younger youth (< 18 years). Similarly, SU brief interventions have largely been developed for adolescent and general adult populations, rather than young adults (Mitchell, Gryczynski, O'Grady, & Schwartz, 2013; Sommers et al., 2013). Studies that have focused on young adults tend to be among college students; SU interventions among JIYA are limited (O’Connor et al., 2018; Tanner-Smith & Lipsey, 2015).

Thus, we sought to develop and test a new service delivery model that integrates evidence-based strategies to move youth through the HIV and SU care cascades via testing/screening, brief intervention to promote service readiness and to reduce risk, and cross-system linkage to increase enrollment in care. We utilize a randomized controlled trial design, with a 1:1 randomization to test the efficacy of the intervention. Building on prior innovations to improve service delivery for JIYA, the model is delivered *within* an alternative sentencing program via a partnership with a community health system to address unmet HIV and SU service needs in JIYA. In the absence of a large body of literature on the organizational predictors of implementing successful cross-system service delivery models within justice settings, key implementation elements of the intervention and its delivery such as uptake, feasibility, acceptability and sustainability (Proctor, Powell, & McMillen, 2013) are also explored. In this paper, we describe the theoretical models and frameworks used to guide the development and implementation of this new integrated service delivery model as well as the study protocol for a randomized controlled trial that is currently underway to test the model.

### The guiding theoretical model for intervention adaptation and framework for implementation

National policies to reduce HIV, such as Treatment as Prevention or Seek, Test, and Treat (Gardner, McLees, Steiner, Del Rio, & Burman, 2011), emphasize the importance of HIV testing as a tool to identify HIV infection, and facilitate linkage to HIV care. The aim of these policies is to effectively move HIV+ persons through the HIV care cascade (e.g. from screening to enrollment in treatment) so as to achieve engagement in treatment and ultimately viral suppression (Dieffenbach & Fauci, 2009; Mugavero et al., 2012; Office of National AIDS Policy, 2020). A similar behavioral health care cascade (Belenko et al., 2017) has been developed which aims to move justice-involved youth from screening and identification of substance use problems in justice settings to treatment initiation and engagement in community behavioral health agencies. These care cascades identify discrete steps that occur in the practice continuum of getting an individual in treatment with the end goal being either viral suppression in the case of HIV or sustained recovery with substance use: screening, assessment, service referral, treatment initiation, and treatment retention. When selecting interventions to adapt and integrate for JIYA, we identified those that would address specific steps in the care cascade. Existing evidence-based interventions were adapted and integrated using Andersen’s Behavioral Model of Health Services Use (Andersen, 1995).

**Fig. 1:**
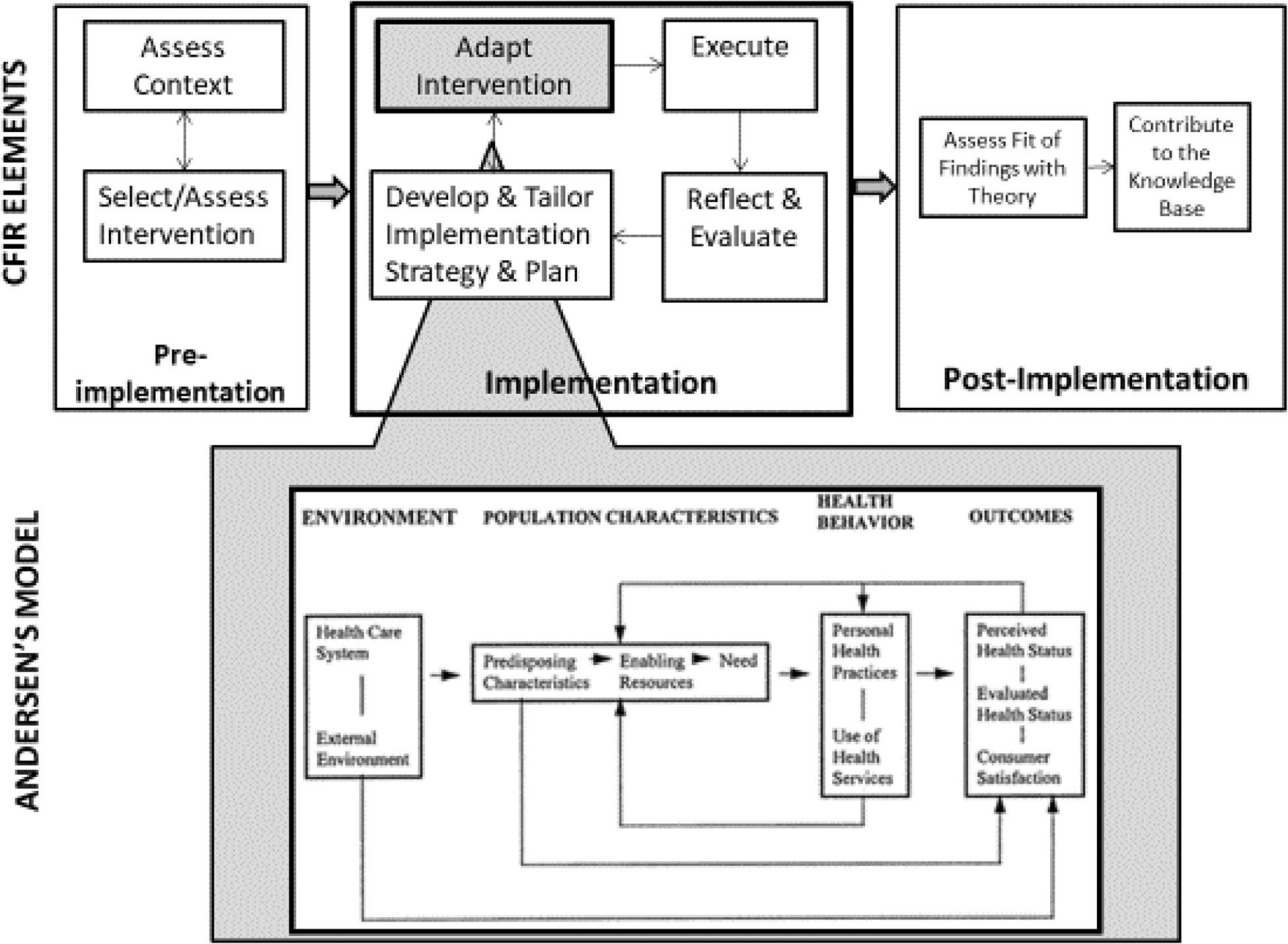
Theoretical frameworks guiding intervention adaptation and implementation

Andersen’s model provides a useful framework for key domains that need to be targeted in a screening and linkage intervention for JIYA so as to promote service uptake. This model, which has been used widely to inform health behavior and health services access and utilization in a variety of populations (Bannon & McKay, 2005; Harrison, McKay, & Bannon, 2004;McKay, Pennington, Lynn, & McCadam, 2001; McKay & Bannon Jr, 2004), highlights environmental (e.g. social contexts), population characteristic (e.g. predisposing characteristics, enabling resources, perceived/evaluated need), and individual health behaviors that influence uptake of health care and other positive health outcomes. Intervention targets include environment and population characteristics that influence change in health behaviors/health outcomes (see Fig. 1).

The Comprehensive Framework for Implementation Research (CFIR) (Damschroder & Hagedorn, 2011) served as the guide for understanding of system/organizational, provider and consumer factors that influence acceptability, feasibility, and potential sustainability of the model. As with many implementation science frameworks focused on achieving practice or process improvement in various settings (Moullin, Sabater-Hernández, Fernandez-Llimos, & Benrimoj, 2015) CFIR emphasizes multi-level influences on program implementation: 1) assessment of context (pre-implementation phase); 2) intervention adaptation, implementation and development of an implementation strategy (implementation phase); and 3) assessment of fit of findings with theory (post-implementation phase). In the pre-implementation and implementation stages, CFIR examines facilitators and barriers: (i) intervention characteristics, (ii) outer setting in which organization is situated, (iii) inner setting (organizational climate and interagency coordination), (iv) characteristics of individuals (staff attitudes, skills); and (v) implementation process (i.e., “change process capability”). CFIR is well-suited for guiding implementation of multi-level service delivery models like the one proposed here (see Fig. 1) (Ruffolo & Capobianco, 2012; Sorensen & Kosten, 2011).

### Study objectives

The overarching goals of the study are to (i) adapt and integrate a set of evidence-based interventions that target key elements of the HIV, STI and SU care cascades (screening/identification, linkage and engagement in care) into a single multifaceted intervention; (ii) to test the efficacy of this service delivery model, as compared to standard of care (SOC), to decrease risk behavior (HIV/STI and SU), facilitate the timely diagnosis or identification of HIV, STI and SU treatment needs, and increase uptake of necessary services among JIYA; and (iii) conduct a preliminary implementation evaluation, including surveys and interviews with healthcare staff and justice staff, of the implementation approach which involved the integration of two systems with potentially differing missions and mandates: justice and community health.

## Materials and methods

This study is a randomized controlled trial (RCT), comprising two phases. In the first phase, we integrated existing evidence-based practices/interventions that targeted relevant elements of the cascade and adapted them for JIYA. In the second phase, following the development of a protocol for delivery, the RCT was initiated during which we tested the efficacy of the new intervention as compared to standard of care (SOC). JIYA will be interviewed at baseline and 3-, 6-, 9- and 12 months post intervention. At Session 1 of the intervention, and at 6 m and 12 m follow-up interviews, participants will be offered HIV and STI testing. During this phase we are also conducting an implementation evaluation. The implementation evaluation will track all steps in implementation and identify young adult-, staff-, and organizational-level factors that influence program feasibility, acceptability, and sustainability in ASP settings.

### Study location and sample

The study is being conducted at an alternative sentencing program (ASP), located in a criminal courthouse in a large city in the northeast. Young adults receive the intervention, on-site at the ASP, administered by embedded health staff trained at a local, hospital-based community program (health agency partner) that serves 13–24 y.o. youth and young adults at risk of contracting or infected with HIV. Services offered include community outreach for HIV and STI testing, PrEP (Pre-Exposure Prophylaxis), STI treatment, family planning, and mental health evaluations. For HIV+ youth, this program provides comprehensive, coordinated HIV, primary, and mental health care.

### Eligibility, recruitment and enrollment

We are enrolling *N* = 450 youth, ages 18–24 years, who are mandated to an ASP. We expect most (72%) to be males, and minority youth (84%), reflecting the demographics of the ASP population. JIYA are eligible to participate if they are 18–24 years old, are enrolled in the ASP, report having engaged in past year unprotected vaginal or anal intercourse, report being HIV negative, and are conversant in English. JIYA are informed of the study by either ASP staff or by study staff including peer recruiters; peer recruiters are members of the research team who were previously enrolled at the ASP. If interested, a peer recruiter or research assistant (RA) explains the study and conducts a brief screen to assess eligibility. If eligible, a baseline interview is scheduled and written informed consent is obtained at that interview by the RA.

### Integrated intervention: MoveUp

**Fig. 2:**
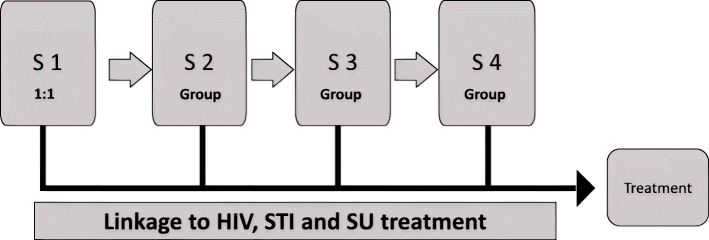
MoveUp Intervention Flow

Screening/identification, risk behavior reduction, linkage and engagement to care were the targets of the intervention and three existing interventions that addressed these elements were selected to be adapted and integrated to meet the needs of the JIYA population and to create a seamless program that would move JIYA through the HIV and substance use care cascades. Based on the extant research, interventions were selected including 1) HIV and STI testing drawn from a model utilized comprehensive care clinic for young adults, developed and maintained by two study investigators (AC, RC); 2) Screening and brief intervention and procedures drawn from NYSBIRT-II (O'Grady et al., 2019) and 3) Mobilizing Our Voices for Empowerment (MOVE), a culturally-based health promotion intervention designed to improve engagement in care and sexual health among HIV+ young Black MSM (Men who Have Sex with Men) through promotion of critical consciousness. Critical media analysis is a central part of the learning and behavior change activities that occur in MOVE (Watts & Abdul-Adil, 1998; Watts, Abdul-Adil, & Pratt, 2002; Watts, Griffith, & Abdul-Adil, 1999; Watts & Guessous, 2006) to enhance critical consciousness, group members are shown media and taught a method of analyzing the media to identify oppressive messages. The integration and adaption process of the intervention was achieved via an iterative process involving feedback from both JIYA and ASP staff. The process will be described in-depth elsewhere (forthcoming); below we describe the final intervention, MoveUp (see Fig. 2).

Intervention sessions are delivered by Health Coaches who were based at the health agency partner and have experience conducting onsite HIV/STI testing and counseling at various community locations around NYC. Health coaches have at least a Bachelors degree and have been trained by the health agency partner to provide pre- and post-test HIV and STI counseling and to administer the tests. As part of their training to deliver MoveUp, Health Coaches also receive Motivational Interviewing training and general group facilitation training. The role of the Health Coach is to deliver the intervention sessions and assist with linkage to services. To enhance engagement with MoveUp the Health Coach who conducts the first session with the youth remains in contact with the youth throughout their involvement in the program via phone/text or in-person, and works to achieve linkage into HIV/STI and/or SU services if necessary.

#### Session 1: health coach 1:1 meeting

Session 1 is a one-on-one meeting with a Health Coach, which lasts approximately 90 min and includes discussion of the JIYA’s sexual risk and substance use behaviors, HIV and STI testing is offered and development of action plans to target risk behaviors. Session 1 begins after the baseline assessment has been completed, between 1 and 7 days. We explain each section of the session below.

(i) *Sexual health assessment*
  To begin, the JIYA and Health coach engage in a conversation about the JIYA’s sexual health, guided by questions that explore condom use, relationship status, partner selection, sexual norms and preferences and sexual history using a MI (Motivational Interviewing) approach that educates and motivates the young adult to reduce sexual risk behaviors and access sexual health services where necessary.
(ii) *HIV and STI testing protocol*
  At the end of the sexual health/risk assessment, participants are offered an HIV and STI test. Prior to the testing offer, as part of the sexual health conversation, the Health Coach ascertains the ability of the JIYA to effectively manage a positive diagnosis. Those who report no social supports or anticipate a negative reaction to a positive test (e.g. articulate harm to self or other) are not offered a test at that session (see testing protocol below). HIV testing is conducted with the *Oraquick™* rapid test. STI testing for chlamydia and gonorrhea are collected using the Gen-probe Aptima Urine Specimen Collection Kit and sent to the New York City Department of Health and Mental Hygiene (NYC DOHMH) for analysis. Participants are informed of their HIV test results during the first session. If the rapid HIV test is reactive, participants receive post-test counseling and immediate referral and linkage for confirmatory testing at the health clinic by the Health Coach; they do not remain in the study. STI testing results are returned within 14 days and delivery of positive results occur via phone. Youth testing positive for STIs will be referred for treatment to the partnering health clinic’s primary care clinic or sexual health clinics established by the NYC DOHMH; JIYA with positive STIs remain in the MoveUp program.
(iii) *Sexual health action plan*
  At the end of the sexual health assessment, JIYA develop action plans related to changing an aspect of their sexual risk behavior (or maintaining healthy behavior for low risk JIYA). Health Coaches help the JIYA specify the goal and identify discrete steps that will assist the youth in achieving the goal. The young adults are offered the opportunity to receive texts reminding them to work on their action plans as well as discuss the progress on their plan with their Health Coach for the duration of the intervention.
(iv) *Substance use risk assessment*
  Following the conclusion of the sexual health assessment, the Health Coach focuses on the JIYA’s substance use behaviors following an adapted version of the *NYSBIRTII* protocol. JIYA receive a SU screen comprising the DAST-10 and AUDIT and are engaged in the adapted BNI (Brief Negotiated Interview). BNI content is tailored to the risk level and specific substance use described by the participant during the screen. Participants scoring in the no/low risk range will receive positive feedback and psychoeducation about SU. Participants scoring in the moderate, harmful, and severe risk ranges will receive feedback on patterns of SU and their effect on health, social, legal/justice and other outcomes and more extensive psychoeducational materials. Following BNI structure, Health Coaches use motivational interviewing tools and techniques to increase awareness of problem use, encourage behavior change, and motivate willingness to seek services. JIYA who score in the harmful or severe risk zones may also be linked via their Health Coach to SU treatment with a community-based provider.
(v) *Substance use action plan*
  At the end of the substance use assessment and conversation, JIYA with the assistance of the Health Coach will develop a second action plan focused on changing an aspect of their substance use behavior (or maintaining healthy behavior for low risk JIYA). The exact same format is followed for the sexual health action plan, including the opportunity to receive texts reminding them to work on their action plans.

#### Sessions 2–4: group intervention

Sessions 2–4 are a group format and occur weekly. In *Session 2* JIYA are introduced to the concept of critical consciousness, they are taught about the connections between oppression, powerlessness, and health risk through analysis of current media (including social media) and discussions about oppressive forces in their daily lives. They are also introduced to the concept of empowerment, and how they can use critical consciousness to make positive changes leading to healthy decisions in their lives, and the lives of friends/family and society.

In *Session 3* JIYAs engage in a series of engaging exercises for practicing basic (cognitive, behavioral and social) skills for: self-awareness; risk perception; goal-setting and decision-making; communication and negotiation; and action-planning related to substance use and sexual risk behavior. Examples of major HIV prevention and substance use foci include: risky sex: healthy vs. unhealthy relationships; sexual negotiation; pros and cons of drugs and alcohol; personal behavioral analysis; and substance use intervention and services. JIYAs also have the opportunity to share their progress on their sexual health and SU action plans created in Session 1, and receive encouragement and tips for success from the Health Coaches and their fellow group members.

In the final session, *Session 4*, young adults continue to build their capacities for positive coping. Examples of major intervention foci include: defining and identifying stress; problem solving skills; self-calming cues; supportive relationships (family support); and discussion of progress and challenges on their action plans. Session 4 also includes a review of the first 2 group sessions and a graduation activity.

#### Linkage

During Session 1 after Health Coaches have worked with participants to identify their HIV, STI and SU risk and service needs, the health coach then tackles JIYA- and systems-level barriers to linkage and engagement in care. Using the same strengths-based approach, health coaches work with JIYA to *begin* to (a) achieve acceptance of his/her HIV/STI risk or treatment needs, and/or problem substance use; (b) identify enabling and predisposing factors to accessing care (perceived and logistical/structural); (c) identify personal strengths to effectively overcome these barriers; and (d) create and execute a plan to overcome barriers to accessing medical care or SU treatment. Completing the steps of the linkage protocol happens over the course of the JIYA’s participation in the intervention (~ 4 weeks) as the Health Coach remains in contact with the JIYA, speaking, texting etc. with the JIYA as required (e.g. daily, weekly) in order to achieve linkage. In this role, the Health Coach also serves as the liaison between JIYA, clinical providers, and ASP staff, and is additionally responsible for facilitating communication, access to insurance if necessary and confirming appointments. Health Coaches also document frequency of sessions and contacts for each JIYA in MoveUp.

### Randomization process and standard of care

#### Randomization

**Fig. 3:**
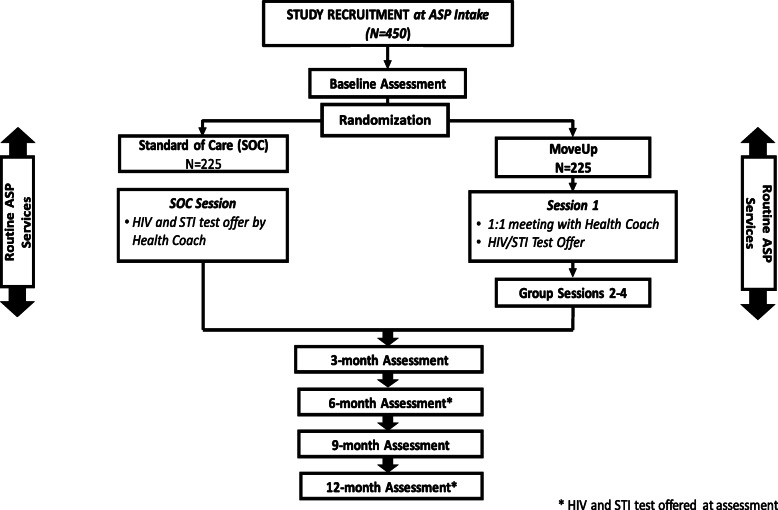
Study Design of MoveUp Randomized Control Trial

Following the baseline interview, a Session 1 is scheduled with the participant and at that session, randomization occurs (1:1) to either the MoveUp or standard-of-care (SOC) condition using randomization schedules based on randomly permuted blocks and stratified by gender to ensure similar distribution in both arms (Fig. 3).

#### Standard of care condition (SOC)

SOC is what study participants receive as part of their standard participation at the ASP. The ASP aims to reduce recidivism via providing or linking to training and assistance in education, vocation/employment, public assistance, Medicaid/insurance, housing, screening and referral to behavioral health programs. Although the ASP has Case Managers on staff, linkage to behavioral health treatment usually only involves referral that includes name and location of the provider/agency; referral to health services has not previously been a component of standard practices at the ASP.

#### SOC HIV and STI testing

At Session 1, if a participant is randomized to SOC they are also offered a rapid HIV test and STI test at that time by the Health Coach. The offer includes an explanation of a) what HIV, chlamydia and gonorrhea are, b) how they are transmitted, and c) testing procedures (i.e., oral swab and urine screen) as is standard in most testing facilities. SOC participants who accept the HIV test and whose test is reactive receive emotional support and post-test counseling, and are linked to HIV care immediately (i.e., referred for confirmatory testing to sites such as the partnering health clinic). STI testing and feedback of results are completed in the same fashion as with JIYA in the intervention arm.

### Primary study outcomes

Three primary study outcomes as assessed at 12 months include (i) HIV/STI risk behavior defined as number of unprotected anal or vaginal sexual occasions in the past 3 months; (ii) acceptance of HIV or STI testing when offered (y/n); and (iii) substance use frequency defined as number of days in past 30 days. *Secondary program outcomes* include (ii) number of youth referred to SU treatment; (iii) and of those, number attending at least one treatment session. Finally, *additional exploratory program outcomes* include (i) the number of HIV+ youth referred to HIV treatment; and of those, the number attending at least one treatment appointment; and (ii) the number of PrEP eligible JIYAs referred to PrEP/medical, and of those, the number attending at least one PrEP/medical care appointment.

JIYA HIV sexual risk and substance use behavior is collected using an adapted version of the AIDS-Risk Behavior Assessment (ARBA) (Donenberg, Wilson, Emerson, & Bryant, 2002; Elkington et al., 2008; Teplin, Mericle, McClelland, & Abram, 2003), a well-validated instrument that assesses substance use in the past 30 days, 3 months, 12 months and life-spanning as well as sexual risk behaviors including condom use, knowledge of partner sexual history and sex while drunk/high. HIV and STI testing will be documented by the research team at the baseline, 6- and 12-month interviews. PrEP referral and uptake, treatment referral and treatment attendance is based on youth report and treatment attendance is confirmed with the youth’s provider, pending youth consent to contact the provider. For youth in the intervention arm, service referral, including PrEP and linkage, will also be gathered from Health Coach linkage documentation.

### Implementation outcomes

Implementation outcomes include feasibility, fidelity, acceptability, and potential for sustainability of MoveUp (Proctor et al., 2011).

#### Feasibility

Feasibility refers to the ease with which an intervention or program can be implemented (Proctor et al., 2011). We have developed a MoveUp feasibility checklist completed by Health Coaches measuring three types of program delivery obstacles: obstacles to JIYA participation (e.g., time, competing priorities); concrete obstacles (e.g., transportation); and site/staffing obstacles (e.g., turnover, time/space constraints).

#### Fidelity

Fidelity refers to the extent to which an intervention or program is implemented in accord with its defining features (Proctor et al., 2011). To assess the relationship between planned and actual implementation, we have designed a checklist-based monitoring system with assessments completed by MoveUp Health Coaches after each Session and each linkage contact to quantify activities completed and next steps. Health Coaches also record new circumstances that may threaten fidelity (e.g., JIYA loss/gain of employment; youth criminal involvement). Weekly fidelity monitoring meetings with Health Coaches to provide structured feedback are conducted by Study Team Investigators.

#### Acceptability and sustainability

Acceptability refers to how well an intervention is received by the target population and the extent to which a new intervention might meet the needs of the target population and organizational setting. Sustainability has been defined in the literature as comprising multiple elements or facets, such as financial and political resources within an institution to support the program, support of the broader community and the strength of relationships between the agency in which the program lies and any partnering agencies which refer or receive program participants (Proctor et al., 2011). Acceptability and sustainability will be assessed quantitatively via staff surveys (see Data sources and Measures; Table 1) and qualitatively via focus groups, one each with Health Coaches and ASP program staff following completion of MoveUp. Guided by CFIR, the implementation focus groups will gather information on staff attitudes and perspectives on integration of MoveUp into ongoing ASP programming; partnerships between the health clinic and ASP staff; relationships with partnering health and SU agencies; changes in job role, performance, or load. Focus groups were chosen to provide the opportunity for staff to discuss the acceptability and potential sustainability of the intervention and implementation approach within the context of their larger agency dynamics.

**Table 1:** Table of study assessments

Domain/Measures	Description/Variables/Psychometric properties
**HIV, STI and SU main study outcomes**	
Sexual risk and substance use behavior	AIDS-Risk Behavior Assessment (ARBA) (Donenberg et al., 2002; Elkington et al., 2008; Teplin et al., 2003)
HIV testing/STI testing	Count of youth who agree to HIV and STI test
Linkage to SU treatment, HIV care, STI care, and PrEP	Count of eligible youth referred for STI care, HIV care, SU treatment or referred for PrEP
**Implementation outcomes**	
Acceptability	Client Satisfaction Questionnaire-8 (Attkisson & Zwick, 1982) Evaluation of Linkage and Referral Services Scale (*staff*) Staff acceptability
Potential sustainability	Program Sustainability Assessment Tool (Luke, Luke, Calhoun, Robichaux, & Moreland-Russell, 2014) (*staff*)
**Andersen’s model: predisposing and enabling factors**	
Mental Health	Brief Symptom Inventory (Derogatis, 1993) Lifetime Incidence of Traumatic Events (Greenwald, Rubin, Russell, & O’Connor, 2002)
Socio-emotional characteristics	Sociopolitical control scale (Zimmerman & Zahniser, 1991) Social support scale (Schulz & Schwarzer, 2003)
Substance use norms and treatment behaviors	Survey of Alcohol and other Drug norms Barriers to Treatment Participation Scale (Kazdin, Holland, Crowley, & Breton, 1997) TCU treatment motivation scale (Simpson & Joe, 1993) Service utilization **–** GAIN-I
HIV/STI testing and knowledge	HIV/STI testing history (Kaiser Family Foundation, n.d.; DiClemente, Brown, Beausoleil, & Lodico, 1993; Mullins, Braverman, Dorn, Kollar, & Kahn, 2010; Peralta, Deeds, Hipszer, & Ghalib, 2007; Tolou-Shams et al., 2007) Perceived vulnerability to HIV HIV knowledge – HIVKQ (Carey & Schroder, 2002) STD knowledge (Yarber, 1985) Prep knowledge and attitudes (Ayala et al., 2013; Eaton et al., 2015; Murphy, 2006)
**CFIR: inner setting and characteristics of individuals/staff**	
Organizational (climate, culture, policies and procedures) and staff characteristics	National Criminal Justice Treatment Practices (NCJTP) survey (Taxman, Young, Wiersma, Mitchell, & Rhodes, 2007) (*staff*)

### Data sources and measures

Young adults are assessed at baseline, 3-, 6-, 9- and 12-months post-intervention. ASP staff are assessed before the launch of MoveUp and 12 and 24 months later. When the MoveUp efficacy trial is complete, we will hold focus group discussions with ASP staff and MoveUp facilitators to examine implementation challenges in-depth.

RAs conduct interviews in private rooms at the ASP, the JIYA’s home or other agreed upon locations (1–2 h sessions); follow-up interviews happen at 3-,6-, 9- and 12-months post-baseline. Participants are offered rapid HIV testing and STI testing at Session 1, 6 and 12-month interviews; the same testing protocol is followed as delivered in SOC. Participants are assured that responses are confidential. Interviewers are trained covering informed consent, confidentiality, reporting requirements, interview content, and testing procedures. Table 1 presents all measures, categorized by main outcomes, Andersen’s Model or CFIR constructs.

### Study power

We conservatively estimate about 15% attrition at 12-month FU (Follow-Up). With a baseline sample of 450 participants, we anticipate a final sample of at least *n* = 382 with complete data for the primary analysis. With 191 participants per group, we will be able to provide 80% power to detect a standardized effect size of .33 or more in the reduction of number of unprotected vaginal or anal sex occasions with Bonferroni correction; this is comparable to other important prevention methods. The standardized effect size of .33 is considered as a relatively small to moderate effect size according to Cohen (Cohen, 1992). Even if actual attrition is greater than expected (e.g. 20%), the detectable standardized effect size will only increase slightly to .34.

### Study hypotheses and data analysis

Intent-to-treat principles will be invoked to guide the analysis of the primary outcomes. Relatedly, three primary hypotheses will be examined: compared to JIYA in SOC, JIYA in the treatment condition (MoveUp) will demonstrate (i) a lower number of unprotected anal and/or vaginal sex occasions in the past 3 months at the 12 month FU; (ii) a greater proportion of HIV testing acceptance at Session 1 (y/n), as well as at 6- and 12- months follow-up interviews; and (iii) fewer days of substance use in past 30 days at the 12 month FU. Stratified randomization of participants to the groups based on their gender requires that the analysis also include gender as a covariate. We will use the general framework of generalized linear models (GLM) to model the longitudinal outcome trajectories and generalized estimating equation (GEE) method to account for within-subject correlation across the five time points (baseline, 3-, 6-, 9-, and 12-months). The general form of the analysis model will be g(μ) = α_0_ + α_1_X + βI + ∑γ_i_T_i_ + ∑δ_i_T_i_I, where g denotes the link function (identity for continuous outcome, logit for binary outcome and natural log for count outcomes) X represents the indicator of female (vs. male), I is the group indicator for MoveUp (vs. SOC), and T_i_ is the indicator for time at 3-month, 6-month, and 12-month evaluation (vs. baseline) for i = 1, 2, and 3. The interaction coefficients δ_i_ are of interest, measuring the difference in the rate of change in outcome across the two treatment groups at each follow-up assessment. We will employ the same analytic approach to examine differences between JIYA in MoveUp and in the SOC condition with respect to other study outcomes.

Finally, we will examine potential mediating/moderating variables from the Andersen and CFIR models on study main outcomes. Following our theoretically informed model (Fig. 1) (Fishbein, Hennessy, Kamb, et al., 2001), we will use Structured Equation Modeling to identify theoretical constructs and pathways that were influential in the intervention. For example, MoveUp may have a more positive effect on acceptance of HIV (and STI) testing among JIYA with greater perceived HIV risk, greater motivation to receive services or positive HIV-testing history. Similarly, MoveUp may have a more positive effect on linkage outcomes when operating in an ASP that is more supportive of concepts perceived as innovative by both ASP and Health Coach staff. Here, organizational culture would appear to modify MoveUp’s impact on young adult service referral and engagement outcomes. We will examine the Lagrange Multiplier and Wald Tests to consider the deletion or inclusion of paths (McDonald & Ho, 2002); ultimately, however, deletion or inclusion of paths will be informed by theoretical underpinnings. Once the model is identified, we will test for group differences between intervention conditions in latent constructs and in the proposed paths between these constructs. This method will allow us to estimate the intervention effects on the constructs directly as well as their relationships to one another (Boomsma, 2000). We will use three goodness-of-fit indices: Bentler-Bonnet’s Normed Fit Index, Bentler-Bonnet’s Non-Normed Fit Index, and the Comparative Fit Index. We will also verify the root mean-square error of approximation (RMSEA) as an index of misfit. Well-fitting models will have fit indices of .90 or higher and < .06 for RMSEA.

### Trial registration, ethics approval, and trial status

The research and ethics presented in this study have been reviewed and approved by the new York State Psychiatric Institute Internal Review Board. The study was registered 11 December, 2017 on ClinicalTrials.gov (NCT03369249) (https://clinicaltrials.gov/ct2/show/NCT03369249). Phase 1 activities have been completed and the evidence-based interventions have been adapted, integrated and pilot tested. Recruitment of main subjects has begun, and the trial will reach completion, tentatively January 2022.

## Discussion

This study is one of the first to systematically develop and test an integrated screen/testing, prevention intervention and linkage-to-care services program (MoveUp), using evidence-based approaches to address the overlapping HIV/STI *and* substance use epidemics in justice-involved young adults. JIYA are at considerable risk for HIV/STIs and SU/D, due to a confluence of individual and contextual factors, including problematic substance use and disorder. Yet their absence from systems of care and treatment in the community makes prevention and intervention difficult, resulting in their continued high risk. Providing on-site services to identify HIV risk and treatment need within justice-settings and to link to services in the community, via health and justice system partnerships, represents an innovation that can capitalize on missed opportunities for engaging JIYA in health care.

Most evidence-based practices for HIV services in justice-involved populations have either been developed for younger youth (Tolou-Shams et al., 2019) or delivered for adults in (locked) correctional settings (Belenko, Hiller, et al., 2013; Belenko, Visher, et al., 2013; Elkington et al., 2016). Recent data from juvenile community supervision agencies noted that approximately two-thirds do not offer any HIV/STI related services (Elkington et al., 2020), either on-site or via referral to community-based partners. The primary focus of court or justice mandates in these settings (Gardner et al., 2019) makes integration and sustainment of HIV-related services challenging as staff must work outside their perceived role in order to provide HIV-related services (Elkington et al., In press; Gardner et al., 2019; Tolou-Shams, Harrison, Conrad, Johnson, & Brown, 2017). The development of partnerships between health agencies and those serving justice-involved populations, such as ASPs, can facilitate the delivery of on-site services via integration of community health workers into these settings. Thus, the current study is designed to evaluate an intervention that increases identification of HIV, chlamydia and gonorrhea, reduces sexual and substance use risk behaviors and promotes access to services and care via a model of cross-system collaboration. Moreover, to our knowledge, the integration of approaches to address the overlapping and synergistic risks of HIV/STI and SU/D so as to move JIYA simultaneously through both the HIV (Gardner et al., 2011) and Behavioral Health (Belenko et al., 2017) care cascades, have not previously been accomplished. It is anticipated that the delivery of on-site, integrated services by Health Coaches will greatly aid our ability to identify and link the hard-to-reach and often overlooked population of JIYA to needed services.

Although developing effective prevention and screening programs is important, ultimately, it is imperative that programs be adopted, integrated, and sustained by justice-involved serving organizations and community-based health partners (Elkington et al., In press). The proposed study will also examine key facilitators and barriers within system, organizational and staff contexts that prevent the implementation and ultimately sustainability of such programming’s routine use. Close attention to these factors will provide preliminary information for future implementation research that will develop optimal strategies for achieving this goal (Belenko, Hiller, et al., 2013; Belenko, Visher, et al., 2013; Rubenstein & Pugh, 2006). In particular, it will provide important information regarding the delivery of a multicomponent, multilevel intervention designed with the goal of increasing uptake and sustainment of HIV-related services in justice settings.

## Data Availability

Not applicable.

## Abbreviations

HIV: Human Immunodeficiency Virus
STI: Sexually Transmitted Infection
SU/D: Substance Use/Disorder
JIYA: Justice-Involved Young Adult
ATN: Adolescent Trial Network
CDC: Centers for Disease Control and Prevention
ASPs: Alternative Sentencing Programs
SBIRT: Screening, Brief Intervention and Referral To Treatment (Model)
MI: Motivational Interviewing
CFIR: Comprehensive Framework for Implementation Research
SOC: Standard of Care
RCT: Randomized Controlled Trial
PrEP: Pre-Exposure Prophylaxis
ASP: Brooklyn Justice Initiatives
MSM: Men who Have Sex with Men
BNI: Brief Negotiated Interview
NYCDOHMH: New York City Department of Health and Mental Hygiene
RA: Research Assistant
ARBA: AIDS-Risk Behavior Assessment
PSAT: Program Sustainability Assessment Tool
FU: Follow-Up
GLM: Generalized Linear Models
GEE: Generalized Estimating Equation
RMSEA: Root Mean-Square Error of Approximation
